# Adaptive Illuminance Effects on Retinal Morphology and Refraction: A Comprehensive Study of Night Myopia

**DOI:** 10.3390/jcm13010211

**Published:** 2023-12-29

**Authors:** Elvira Orduna-Hospital, Cynthia Crespo-Castan, Francisco J. Ávila, Ana Sanchez-Cano

**Affiliations:** Department of Applied Physics, University of Zaragoza, 50009 Zaragoza, Spain; 679599@unizar.es (C.C.-C.); avila@unizar.es (F.J.Á.); anaisa@unizar.es (A.S.-C.)

**Keywords:** retinal shape, retinal thickness, night myopia, optical coherence tomography, aberrometry, adaptive illuminance

## Abstract

Background: We aimed to study the optical and retinal modifications that occur after adapting to different lighting conditions including photopic, mesopic, scotopic, blue light and red light conditions. Methods: Thirty young healthy subjects with a mean age of 23.57 ± 3.45 years were involved in the study (both eyes included). They underwent aberrometry and optical coherence tomography at both the central and peripheral retina with the 3 × 3 mm^2^ macular cube protocol before starting adaptation to the illuminations (baseline) and after remaining for 5 min under the five different lighting conditions inside a controlled lighting cabinet. Results: Significant myopization (*p* = 0.002) was observed under scotopic and mesopic lighting conditions, while hypermetropization occurred under the influence of blue LED light. In the central retina, a significant thickening of the inner temporal (*p* = 0.025) and outer inferior (*p* = 0.021) areas was observed in the scotopic area, and the thickening increased even more under blue and red light. The mean central thickness decreased significantly under photopic lighting conditions (*p* = 0.038). There was an increase in the mean volume of the central retinal area with red light and a reduction in the volume under photopic lighting (*p* = 0.039). In the peripheral retina, no significant thickness changes were observed after adapting to any of the lighting conditions (*p* > 0.05). Regarding morphological changes, a significant increase in retinal eccentricity (*p* = 0.045) and the shape factor (*p* = 0.036) was found. In addition, a significant correlation was found only between the eccentricity and volume of the central retina in scotopic conditions (r = −0.265; *p* = 0.041), meaning that a higher volume was associated with lower retinal eccentricity. Conclusions: When exposed to different lighting conditions, the retina changes in shape, and ocular refraction is modified to adapt to each condition, revealing the phenomenon of night myopia when transitioning from photopic to scotopic regimes.

## 1. Introduction

Night myopia, a subject of study and controversy for over two centuries, involves an alteration in ocular refraction under reduced lighting conditions compared to photopic lighting [1]. It results in an increase in the eye’s refractive power when transitioning from daytime to nighttime lighting, which has been studied for years [2]. Accommodative errors seem to be the primary cause of this phenomenon, with an average value of approximately −1.50 D, significantly affecting image sharpness when uncorrected [3].

Early investigations suggested an association between night myopia and increased spherical aberration and accommodation. However, the exact contribution of these factors to myopic defocus remains unclear [4]. The Purkinje shift, known as the shift from photopic sensitivity peaking at 555 nm to scotopic sensitivity at 509 nm, occurs due to the transition from cone-based to rod-based responses in the eye [5]. This shift is also linked to an increase in the spherical aberration due to pupillary dilation, refracting the rays that enter through the peripheral pupil before those that pass through the center of the pupil. The role of the peripheral zone, moreover, is favored by the decrease, or even absence, of the Stiles–Crawford effect, which relates the visual luminous efficiency to the directionality of the cones in the retina, reaching the maximum in the fovea [3].

In terms of chromatic aberration, shorter wavelengths focus in front of the retina, causing retinal sensitivity to shift toward those regions in low light. The eye also maintains partial focus during low-light conditions, contributing to a myopic shift in dim light conditions [6]. Some studies that utilized cycloplegics revealed a subsequent reduction in night myopia, emphasizing the role of accommodation [7], while others reported that the causes of this night myopia were spherical and chromatic aberration [4]. The conclusion on the role of accommodation in nocturnal myopia after cycloplegia use was inconclusive. The paralysis of accommodation resulted in changes in normal spherical aberration, suggesting a link to reduced night myopia in such conditions [7,8]. Additional studies highlighted spherical aberration as another cause of eye myopization, showing changes when exposed to an accommodative stimulus versus a point source on a dark background, simulating night vision conditions [8].

In addition, the association between retinal macular thickness and its shape with refractive errors has been verified. In the case of myopia, there is a reduction in thickness in the peripheral part, while the thickness of the fovea, as well as its curvature, increases [9,10,11]. It has also been shown that the choroid, which is the vascular layer that nourishes the retina, plays an important role in refractive errors, especially myopia. A thinning of the choroid is involved in the development and progression of myopia due to the increase in the axial length [12].

Numerous studies highlight the significant impact of light on refraction changes, particularly how reduced light intensities lead to increased myopic refractive errors [13,14,15,16,17,18]. It has been demonstrated that the choroidal thickness varies significantly based on light exposure, influencing the retinal shape [19].

Research findings have emphasized the impact of light exposure on retinal thickness and myopia progression. More light exposure, such as that occurring during the summer, can slow myopia progression and even reduce ocular growth, especially in the vitreous chamber and axial length [13,14,15,16,20]. Furthermore, one’s geographical location is linked to the visual system, with reduced myopic progression in areas with more hours of sunshine and outdoor activities [13,14,15,16,20].

Both refraction and retinal thickness and morphology can be modified depending on the environmental lighting to which an individual is subjected. In this sense, the main objective of this work was to study ocular refractive and retinal morphological changes, both central and peripheral, when adapting for a short time to different lighting conditions (photopic, mesopic, scotopic, blue light and red light). In addition, the increase in myopia was assessed when light conditions were reduced and if the retinal shape underwent any change in morphological readaptation in the peripheral field correlative to the phenomenon of nocturnal myopia.

## 2. Materials and Methods

### 2.1. Sample Selection

This research was carried out at the Department of Applied Physics of the University of Zaragoza (Spain), following the principles established in the Declaration of Helsinki, and after approval by the Clinical Research Ethics Committee of Aragon (CEICA) under reference number PI21-074. The participants underwent continuous measurements in the same session, reaching a total of 60 eyes. All measurements were performed by the same investigator who informed the participants about the study and asked them to sign the informed consent form if they agreed.

All healthy young subjects of legal age with refractive errors less than 4.00 D of sphere and 3.00 D of cylinder were included in the study, with the following exclusion criteria: individuals with accommodative or binocular problems, amblyopia or strabismus; those with visual acuity less than 0.80 in either eye; individuals with ophthalmic or systemic pathologies affecting vision; opacification of ocular media; those who had used electronic devices one hour prior to measurements; and individuals who had consumed coffee or alcohol within two hours of the experiment or had smoked, as these factors can affect retinal function. Participants were asked to wear ophthalmic lenses and remove them during the study to prevent the lenses from filtering out any wavelength of light to which they were going to adapt and from thereby falsifying the results of the experiment.

### 2.2. Cabinet with Controlled Lighting Conditions and Instruments

**Figure 1:**
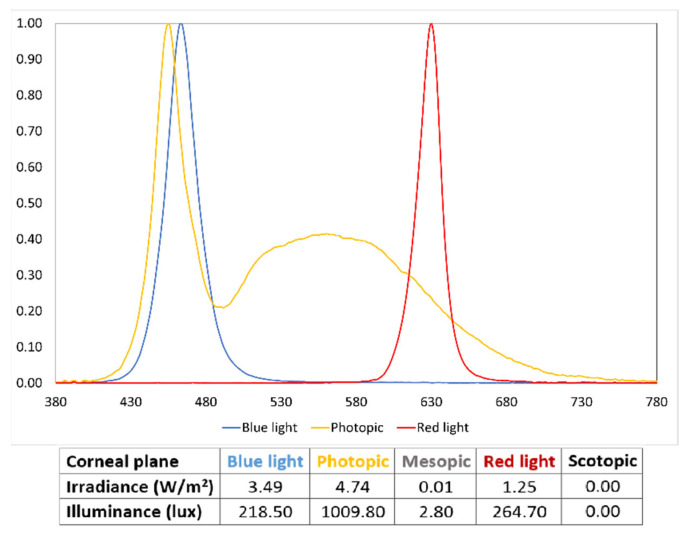
Normalized spectral irradiance (W/m^2^/nm) of the light that reaches the corneal plane with each illumination. Blue light peaks at 463 nm and red light at 630 nm; mesopic lighting was obtained, dimming the photopic spectra.

The experiment was carried out inside a custom-made controlled lighting cabinet that was in a room under scotopic conditions. The lighting system featured two LED luminaires positioned at the top of the cabinet, while the walls were finished with the standard internal Munsell N5 color. It was controlled using an 8th generation iPad Model A2270 (Apple Inc., Cupertino, CA, USA), configured with five lighting conditions (photopic, mesopic, scotopic, blue light and red light). Illumination values at the corneal plane were measured using a StellarNet Black-Comet spectroradiometer, maintaining the same conditions throughout the study (Figure 1).

The instrument used to measure refraction was a commercial clinical aberrometer IRX3 Shack-Hartmann (Imagine Eyes, Orsay, France), which automatically calculates the aberrations that are adjusted to the corresponding wavefront. Six measurements were made, all under scotopic lighting conditions: a first initial baseline measurement upon arrival of the subject and a measurement immediately after the subject had adapted for 5 min to each type of lighting (photopic, mesopic, scotopic, blue light, and red light). Aberrometric data with a 4 mm pupil were exported and categorized with respect to the illumination, eye, and person to add to the database.

**Figure 2:**
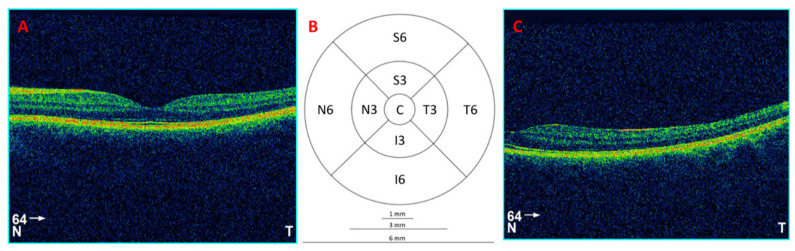
(**A**) Tomographic image of the central retina from a 64-scan of a left eye (LE). (**B**) The 9 areas of the ETDRS grid: central or subfoveal area of 1 mm diameter (C), internal temporal area (T3), internal nasal area (N3), internal superior area (S3), internal inferior area (I3), external temporal area (T6), external nasal area (N6), external superior area (S6) and external inferior area (I6). (**C**) Tomographic image of the peripheral retina belonging to 64-scan LE.

An optical coherence tomography (OCT) device was used to explore the retina, specifically the 3D OCT-1000 (Topcon Corporation, Tokyo, Japan), and its 3 × 3 mm^2^ macular cube protocol centered around the fovea, performing 128 B-scans. This protocol provides data for total retinal volume and retinal thickness divided into the Early Treatment of Diabetic Retinopathy Study (ETDRS) grid with three concentric circles with a diameter of 1 mm, 3 mm (inner ring) and 6 mm (outer ring), the latter two divided into four quadrants: superior, temporal, inferior and nasal (Figure 2).

First, the subject was asked to look at the central fixation stimulus (a green square) to obtain an image of the central retina, and then the internal fixation was moved horizontally 15° to the right for the right eye (RE) and 15° to the left for the left eye (LE) to acquire the image of the temporal peripheral retina. This process was repeated for each eye of each individual six times, as was stated with the aberrometer. The macular maps were automatically segmented using the device’s software, taking the retinal thickness from the internal limiting membrane to Bruch’s membrane. At all times, it was verified that the scans were of high quality and that the retinal segmentation was correct, discarding the defective scans. The values of the retinal thicknesses of the 9 quadrants of the ETDRS of the central and peripheral retina were exported manually. The images of each of the 128 scans that had been made in each measurement were also exported. The data classification was performed in a similar way to the aberrometer with the addition of the two fixation positions.

### 2.3. Image Processing Algorithm

**Figure 3:**
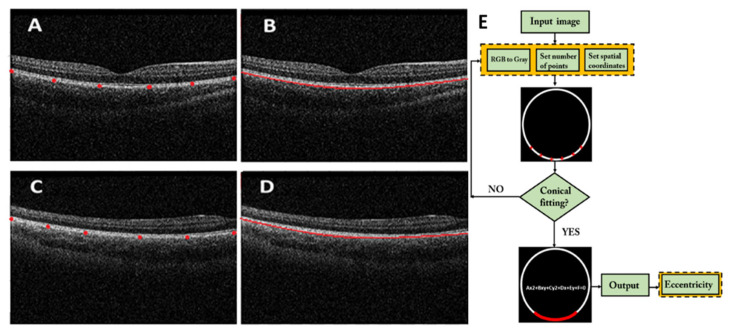
The 6 points established in the pigmented epithelium of the central (**A**) and peripheral (**C**) retina in the 64-scan, with its corresponding conical segmentation marked with the red line (**B**,**D**). Schematic diagram of the proposed segmentation algorithm (**E**).

The analysis of retinal shape was carried out using a custom-written image segmentation algorithm [21] in MATLAB programming language (R2020a, Mathworks, MA, USA) to automatically detect the retinal pigment epithelium from OCT images, and after contour detection, the best conical fitting was computed to extract the asphericity, eccentricity and shape factor (SF) parameters. Then, the retinal conic equation was computed for each subject and lighting condition. This was performed for both the central and the peripheral retina using the 64-scan of each measurement. It was decided to take 6 points of the retinal pigmented epithelium (RPE) as spatial coordinates for the calculation, after reliability tests of the parameter taken with a greater number of points, resulting in a negligible difference if 12 points were taken instead and then reducing the computational costs and image-processing time. If the conical equation could not be fitted with the software (due to a loss of local contrast, for instance), the program would indicate the need to change the number of spatial coordinates required, ensuring the minimum number of artifacts and calculation errors. Figure 3 shows an example of the software operation at the foveal location (Figure 3A,B) and at the peripheral retina (Figure 3C,D). Figure 3E depicts the flux diagram of the proposed algorithm, which first converts the RGB OCT images to grayscale and then starts the detection of the RPE border throughout 6 spatial coordinates, from which it finally computes the conical equation for extracting the mentioned shape parameters (i.e., eccentricity, asphericity and SF).

### 2.4. Measurement Protocol

The protocol began with baseline measurements using an aberrometer and OCT, with subjects not exposed to any prior light stimulation. Participants, without wearing glasses or contact lenses, were positioned with their eyes opened and their heads inside the cabinet, leaning on a chin rest to maintain consistent lighting and prevent variations due to head movements. Each subject spent 5 min in each illumination condition, with no other stimuli visible apart from the cabinet walls, ensuring they could not look directly at the light source. Following each adaptation to different lighting conditions, aberrometry and OCT assessments were conducted in a random order for each participant to prevent measurement bias.

### 2.5. Statistical Analysis

Data collected in the study were recorded on Excel databases (Microsoft^®^ Office Excel 2019, Microsoft Corporation, Redmond, WA, USA) and analyzed with the Statistical Package for the Social Sciences (SPSS 24.0 Inc., Chicago, IL, USA). First, descriptive statistics were computed for quantitative variables, including the mean, standard deviation (SD), maximum and minimum values. The normality of the data distribution was assessed with the Kolmogorov–Smirnov test, revealing nonnormality. To compare variables such as refraction and retinal morphology under different lighting conditions, nonparametric tests for related samples (specifically, Friedman’s test) were employed. Statistical significance was defined as a *p* value less than 0.05.

## 3. Results

Of the 60 healthy young eyes included, 40 were female and 20 were male. The mean age of the subjects was 23.57 ± 3.45 years, ranging from 20 to 34 years. The mean spherical equivalent (SE) was −1.31 ± 1.83 D and ranged from −4.00 D to +2.25 D.

### 3.1. Refractive Errors

**Figure 4:**
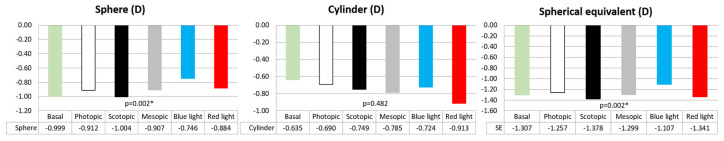
Bar graphs of the refractive errors (sphere, cylinder and spherical equivalent) measured with the aberrometer in the baseline measurement and after adapting to the different lighting conditions (photopic, scotopic, mesopic, red light and blue light), as well as the statistical significance among them (*p* value). A *p* value less than 0.05 is marked with an asterisk (*).

Significant differences were obtained when comparing the refractive errors obtained after adaptation to the different lighting conditions for the sphere (*p* = 0.002) and for the SE (*p* = 0.002). In both cases, myopia tended to occur in scotopic lighting conditions, followed by the basal measurement and mesopic light, while myopia decreased considerably with exposure to blue light (Figure 4). No significant differences (*p* = 0.482) were found for astigmatism.

### 3.2. Retinal Thickness and Volume

**Figure 5:**
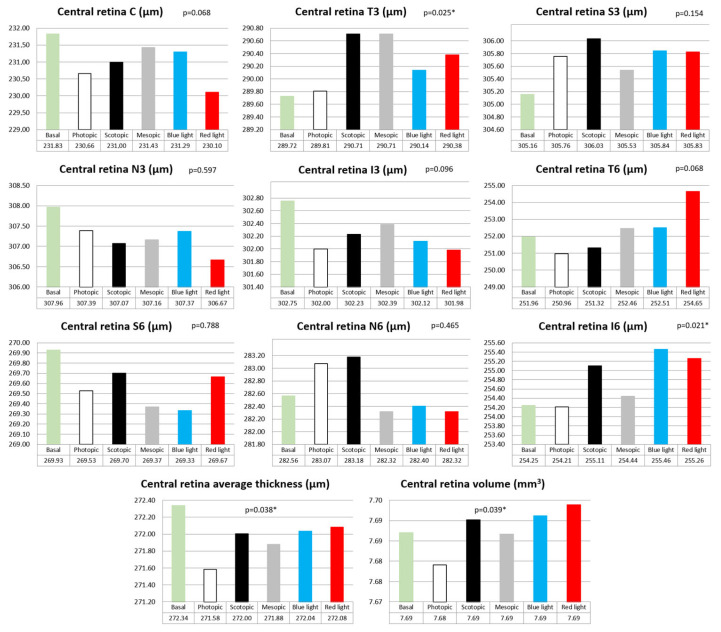
Bar graphs of central retinal thicknesses with the different lighting conditions for each ETDRS area, for the average thickness and for the total volume, as well as their statistical significance (*p* value). Central area (C), internal temporal area (T3), internal nasal area (N3), internal superior area (S3), internal inferior area (I3), external temporal area (T6), external nasal area (N6), external superior area (S6) and external inferior area (I6). A *p* value less than 0.05 is marked with an asterisk (*).

When comparing the central retinal thicknesses measured with OCT in the nine ETDRS areas between the baseline measurement and the five lighting conditions, there were significant differences in thickness in the temporal inner area (T3, *p* = 0.025), with increased thickness under scotopic and mesopic light conditions, and in the lower external area (I6, *p* = 0.021), also with increased thickness in scotopic conditions, but even more so with blue and red light conditions (Figure 5). In both cases, the smallest thickness was obtained under photopic lighting conditions.

Statistically significant differences were also found in the average thickness (*p* = 0.038), with the highest value in the basal measurement and the lowest in photopic lighting conditions. Statistically significant differences were also seen in the volume (*p* = 0.039), with the maximum volume in red light and the minimum volume in photopic lighting conditions (Figure 5).

**Figure 6:**
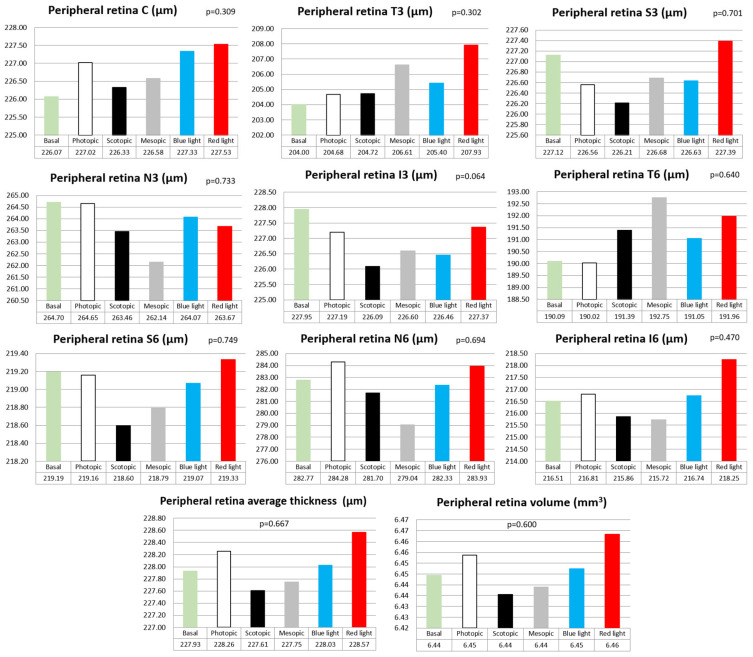
Bar graphs of peripheral retinal thicknesses with the different lighting conditions for each ETDRS area, for the average thickness and for the total volume, as well as their statistical significance (*p* value). Central area (C), internal temporal area (T3), internal nasal area (N3), internal superior area (S3), internal inferior area (I3), external temporal area (T6), external nasal area (N6), external superior area (S6) and external inferior area (I6).

In contrast, in the peripheral retina, a generalized increase in thickness was observed in all areas, in the average thickness and in the volume for lighting conditions with red light, but there were no statistically significant differences between lighting conditions in any case (*p* > 0.05) (Figure 6).

### 3.3. Retinal Shape

**Figure 7:**
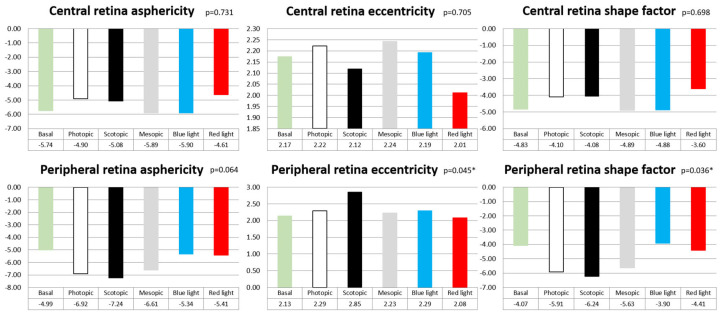
Bar graphs of the ellipticity parameters evaluated (asphericity, eccentricity and SF) using the 64-scan of the central retina and the peripheral retina for the different lighting conditions, as well as their statistical significance (*p* value). A *p* value less than 0.05 is marked with an asterisk (*).

When assessing the retinal asphericity, eccentricity and SF across various lighting conditions to examine retinal alterations, significant changes were observed in the peripheral retina, with differences in eccentricity (*p* = 0.045) and SF (*p* = 0.036), while no variations were detected in the central retina’s shape parameters under the different lighting conditions. (Figure 7).

**Figure 8:**
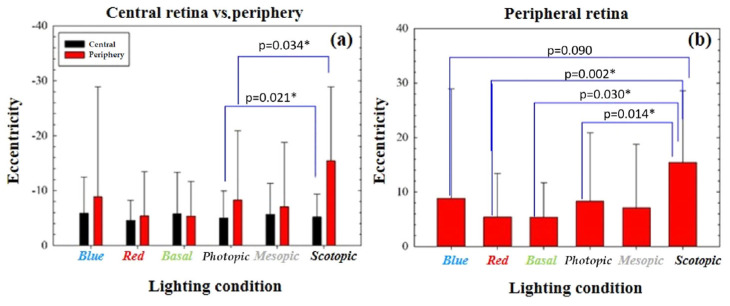
(**a**) Comparison of eccentricity in the central retina vs. peripheral retina under different lighting conditions. (**b**) Comparison of retinal eccentricity when going from one illumination to another. A *p* value less than 0.05 is marked with an asterisk (*).

Significant differences were observed when comparing the eccentricity in the central retina vs. peripheral retina under both photopic (*p* = 0.021) and scotopic (*p* = 0.034) conditions. The retina flattens (greater hyperbola, greater radius) when moving from the central to the peripheral zone (Figure 8a). A significant change in eccentricity was also observed when comparing the retinal shape with red light (*p* = 0.002), photopic (*p* = 0.014) and basal (*p* = 0.030) illumination with that obtained in scotopic conditions, excluding mesopic conditions (*p* = 0.232) and blue light (*p* = 0.090) (Figure 8b).

When examining the eccentricity of the peripheral retina under different lighting conditions, the highest degree of curvature (2.08) was observed with red illumination, followed by basal (2.13), mesopic (2.23), photopic, and blue (2.29) lighting, while scotopic lighting resulted in the least peripheral curvature (2.85). A comparison such as the one mentioned was not conducted for asphericity or SF, as changes in eccentricity were expected to correspond with changes in these parameters due to their correlated nature.

Regarding the relationships between the analyzed parameters, a significant positive correlation was found only between the eccentricity of the central retina and the total volume of the retina in scotopic conditions (r = −0.265; *p* = 0.041), noting that the larger the volume was, the lower the retinal eccentricity. The rest of the correlations between the retinal thickness, volume and eccentricity in the different lighting conditions were not significant.

## 4. Discussion

In this study, our primary objective was to conduct a comprehensive analysis of retinal behavior by thoroughly evaluating the central and peripheral retinal morphology in healthy young adults following brief exposures to a variety of distinct lighting conditions, while also seeking to identify potential refractive changes and examine the retinal shape. The analysis of these parameters as a whole provides a comprehensive understanding of the influence that the different evaluated lighting conditions have on ocular behavior, particularly on the retina.

In the present study, we were very rigorous with the lighting conditions to avoid bias. Myopic SE values are reported to be increased in both scotopic (−1.38 ± 2.10 D) and red light (−1.34 ± 2.14 D) conditions, and decreased in mesopic (−1.30 ± 1.83 D), photopic (−1.26 ± 1.87 D) and blue light (−1.11 ± 1.93 D) conditions, with significant differences in scotopic conditions (*p* = 0.002) according to other studies [2,22,23]. It has been previously described that in scotopic conditions human responses to stimuli have been observed to resemble those in myopia, leading to the use of more negative optical aids than typically needed in photopic conditions, independent of the pupil size [2]. A similar study was carried out with phakic and aphakic subjects, observing the myopization of the central retina in scotopic conditions, which was only significant in phakic subjects; these results also did not vary with miosis or pupillary mydriasis [24]. In the present study, a significant refractive change to myopization with nonaccommodative scotopic stimuli was demonstrated. Comparing our results in scotopic conditions with the basal ones, a myopization of −0.071 D was obtained when switching to scotopic vision. Epstein et al. [25] noted that exposing the eye to various lighting conditions, including mesopic, photopic and scotopic conditions, manifested so-called magna myopia, prompting the consideration of this scotopic myopia detection or correction in specific subjects. In contrast, the eye became considerably hyperopic when exposed to blue light, increasing by +0.1996 D. These results are partially supported by those of a study in chickens under a blue light and lens-correction combination, which led to an improvement in the interventions to protect against myopia development with this pattern [26], although some authors suggest that ocular longitudinal chromatic aberration (LCA), which causes wavelength defocus and differing refraction between short- and long-wavelength light, may explain the wavelength-dependent refractive error found [27]. Chirre et al. [28] observed that, on average, the monocular accommodation error when luminance decreases tends toward the average dark focus, producing nocturnal myopia, being lower under binocular vision, which means that binocularity mitigates nocturnal myopia when vergence accommodation is mediated.

Our findings indicate that short-term exposure to different lighting conditions has distinct effects on the variation in both the retinal thickness and volume, suggesting that the light wavelength serves as a specific target in these effects. This fact was previously described in the choroidal thickness under blue- or red-light stimuli [19], as these authors pointed out, and while it may be speculative due to the short-term nature of the exposure in our study, it hints at the possibility that varying wavelengths exert distinct impacts on ocular development. In connection with this matter, there have been seasonal studies that uncovered a higher prevalence of myopia during the winter, a season characterized by a reduced exposure to sunlight. This trend may be linked to observed alterations in ocular structure, possibly caused by decreased outdoor activities and diminished sunlight during the winter months, specifically correlating with an increase in the depth of the vitreous chamber and axial length [13,14,20]. The increase in the vitreous chamber could be potentially associated with reduced retinal thickness, especially in both central and peripheral retinal average thickness, concerning the RPE under scotopic conditions. This suggests that prolonged exposure in this lighting condition may lead to ocular elongation and refractive development, which is supported by an experiment where irradiance and illuminance at the corneal plane and luminance of the reading screen were controlled while performing the task. The observed changes in the retina were linked to anterior pole alterations during accommodation, persisting in the retina with some stability after 21 min of reading compared to the baseline condition under appropriate photopic lighting settings [29]. In this line, a study in which a comprehensive analysis of the impact of reading on electronic devices under a range of lighting conditions was conducted [21] revealed that the peripheral retina exhibited varying thickness modifications in comparison to the central retinal area following short reading sessions. This intriguing finding implied a level of retinal instability that appeared to be particularly responsive to daily environmental stimuli. These findings highlighted the dynamic characteristics of the retina and prompted inquiries regarding the possible enduring effects of regular electronic device use, particularly in fluctuating lighting conditions; this was an aspect we examined in our current study, which also identified analogous changes even with different spectra of light and illuminance levels at the corneal plane.

In a recent longitudinal study, compelling evidence suggested that the peripheral retina, notably the superior area, underwent morphological changes, particularly in response to myopia progression. This greater relative myopia in the superior retina could be an antecedent to myopia in initially emmetropic children. Even a minor degree of myopic defocus (approximately 0.3 D), or other factors such as engagement in near-work tasks and accommodation lag, might induce axial length growth and influence the development of myopia. Furthermore, it remains unclear whether these changes result from ocular growth during myopia development. It is conceivable that myopia could start developing at the superior retina, gradually extending to the posterior eyeball, largely influenced by genetic factors. Notably, when emmetropic individuals transitioned to myopia, their ocular shape tended to change more in terms of height than width; these findings shed light on the complex interplay of factors in myopia development, particularly in the superior retina [30]. In our current study, the superior thickness was not the area most influenced by the lighting conditions evaluated, and the results revealed significant differences in eccentricity in both photopic and scotopic conditions when comparing the central and peripheral retina, leading to a reduced peripheral retinal curvature at the analyzed horizontal plane. Significantly, the central retina exhibited an increased curvature under mesopic conditions, followed by photopic and scotopic conditions. The SF exhibited changes, increasing in mesopic conditions and decreasing in photopic conditions, before further decreasing in scotopic conditions. These findings are equivalent to previous results, which showed that retinal eccentricity undergoes substantial modifications in mesopic conditions, resulting in a more curved shape as one moves away from the retinal center, transitioning from a parabolic to a hyperbolic form [21].

Additionally, correlations among the evaluated parameters were analyzed by Breher et al. [31]; they described the relationship between the axial length of the myopic eye and its influence on the retinal curvature radius, obtaining a significant correlation as a result. In addition, they also observed that the horizontal and vertical retinal shapes did not undergo equal changes with the increase in myopia. However, in the present study, the only significant correlation was obtained between the central retina and the total volume (*p* = 0.041) in scotopic conditions, when the higher the volume was, the lower the retinal eccentricity was.

The authors have identified a lack of consistency in the reporting of technical lighting characteristics in studies related to lighting, and this inconsistency not only complicates the comparison of research findings but also hinders the evaluation of the protocols used. A limitation of the present study is that measurements were solely captured at two specific time points: before and after a brief 5-min period of light exposure. Consequently, we lacked information about the temporal progression of the observed impacts on the evaluated parameters. It remains unclear whether changes can manifest within such a short exposure time and whether these changes may evolve over longer durations. Another limitation pertains to the light sources used for different light exposure conditions, as they were not adjusted to achieve uniform luminance. As a result, the effects observed in this study are likely attributable to the specific wavelengths of light. To gain a more comprehensive understanding of the underlying mechanisms, it would be necessary to measure responses to a range of wavelengths and light intensities, a task that falls beyond the scope of the present study.

## 5. Conclusions

In summary, this study revealed statistically significant refractive changes, indicating a trend toward myopization under scotopic, mesopic, and baseline lighting conditions, while hypermetropization was observed under blue light exposure. Morphological changes were primarily observed in the central retina, accompanied by a reduction in curvature when transitioning from the central to peripheral retina in photopic and scotopic conditions. The peripheral retina exhibited the greatest curvature under red light, followed by baseline, mesopic, photopic, blue and scotopic lighting. Moreover, significant increases in eccentricity and SF were noted in the peripheral retina, suggesting potential correlations with alterations in sphericity. These findings have implications for understanding nocturnal myopia and suggest that retinal readaptation effects depend on short changes in ambient lighting.

## Data Availability

The data sets of the current study are available from the corresponding author upon reasonable request.
